# Acid Sphingomyelinase and Ceramide Signaling Pathway Mediates Nicotine-Induced NLRP3 Inflammasome Activation and Podocyte Injury

**DOI:** 10.3390/biomedicines13020416

**Published:** 2025-02-09

**Authors:** Mohammad Atiqur Rahman, Sayantap Datta, Harini Lakkakula, Saisudha Koka, Krishna M. Boini

**Affiliations:** 1 Department of Pharmacological and Pharmaceutical Sciences, College of Pharmacy, University of Houston, 4349 Martin Luther King Blvd, Houston, TX 77204, USAhariniilakkkakula@gmail.com (H.L.); 2 Novi High School, Novi, MI 48375, USA; 3 Department of Pharmaceutical Sciences, Irma Lerma Rangel College of Pharmacy, Texas A & M University, Kingsville, TX 78363, USA

**Keywords:** podocyte, inflammasome, ceramide and acid sphingomyelinase

## Abstract

**Background:** Recent studies have shown that Nlrp3 inflammasome activation is importantly involved in podocyte dysfunction induced by nicotine. The present study was designed to test whether acid sphingomyelinase (Asm) and ceramide signaling play a role in mediating nicotine-induced Nlrp3 inflammasome activation and subsequent podocyte damage. **Methods and Results:** Nicotine treatment significantly increased the Asm expression and ceramide production compared to control cells. However, prior treatment with amitriptyline, an Asm inhibitor significantly attenuated the nicotine-induced Asm expression and ceramide production. Confocal microscopic and biochemical analyses showed that nicotine treatment increased the colocalization of NLRP3 with Asc, Nlrp3 vs. caspase-1, IL-1β production, caspase-1 activity, and desmin expression in podocytes compared to control cells. Pretreatment with amitriptyline abolished the nicotine-induced colocalization of NLRP3 with Asc, Nlrp3 with caspase-1, IL-1β production, caspase-1 activity and desmin expression. Immunofluorescence analyses showed that nicotine treatment significantly decreased the podocin expression compared to control cells. However, prior treatment with amitriptyline attenuated the nicotine-induced podocin reduction. In addition, nicotine treatment significantly increased the cell permeability, O_2_ production, and apoptosis compared to control cells. However, prior treatment with amitriptyline significantly attenuated the nicotine-induced cell permeability, O_2_ production and apoptosis in podocytes. **Conclusions:** Asm is one of the important mediators of nicotine-induced inflammasome activation and podocyte injury. Asm may be a therapeutic target for the treatment or prevention of glomerulosclerosis associated with smoking.

## 1. Introduction

Cigarette smoking is a primary contributor to preventable illnesses and premature mortality on a global scale [1]. Cigarette smoking causes approximately 5.4 premature deaths worldwide [2]. There is substantial clinical evidence suggesting that cigarette smoking can be a significant risk factor for the progression of chronic kidney disease in individuals with diabetes and hypertension [3,4,5]. Moreover, patients who have undergone renal transplantation may experience a decline in renal function due to smoking [6,7,8]. Tobacco smoke is a complex and reactive mixture composed of around 7000 chemicals [9]. The major addictive component in tobacco is nicotine, which stimulates neurons in the ventral tegmental area, leading to dopamine release and reinforcing tobacco addiction. This process involves an increase in excitatory glutamatergic activity onto dopamine cell bodies in the ventral tegmental area [10]. Nicotine exerts a substantial influence on the cardiovascular system, resulting in elevated heart rate and blood pressure, consequently heightening the susceptibility to cardiovascular conditions like heart disease, myocardial infarctions, and stroke [11]. Several studies have already proved that nicotine could damage kidney cells and contribute to the progression of different diseases [12,13]. However, the mechanisms of nicotine-induced kidney damage are still unclear.

The NLRP3 inflammasome functions as a critical sensor involved in both inflammatory and non-inflammatory responses [14,15,16,17,18]. Its role has been well documented in various pathological conditions, including diabetes, silicosis, obesity, gout, chronic kidney disease, acetaminophen-induced liver toxicity [19,20,21,22,23,24,25,26], unilateral ureteral obstruction [27,28], nicotine-induced atherosclerosis [29], kidney injury caused by acute ischemia followed by reperfusion [30], non-diabetic kidney disease [28], and obesity-induced glomerular injury [14]. Additionally, the NLRP3 inflammasome can be activated by diverse stimuli such as bacterial toxins [31], monosodium urate crystals [23], cholesterol crystals [32], ATP, β-amyloid [33], visfatin [34,35], and muramyl dipeptide [36]. Recent studies have implicated nicotine in promoting podocyte injury through NLRP3 inflammasome activation [13]. However, it remains unknown how nicotine triggers NLRP3 inflammasome activation and leads to podocyte injury.

Acid sphingomyelinase (Asm) is an enzyme which leads to the hydrolysis of sphingomyelin to ceramide and phosphorylcholine [37,38]. Ceramide, a bioactive lipid, has been linked to the development of various common diseases, primarily by triggering cell death signaling pathways [39]. One of the enzymes in a group capable of breaking down sphingomyelin is acid sphingomyelinase. This enzyme facilitates the cleavage of the phosphorylcholine linkage in sphingomyelin, resulting in the production of ceramide [40]. ASM may play a crucial role in the regulation of cell and organ functions, potentially contributing to various health conditions within the body, including obesity, diabetes, atherosclerosis, kidney diseases, and disorders in lipid metabolism [38,40]. Therefore, reducing the activity of Asm and decreasing ceramide levels could potentially have a significant impact in diminishing receptor-triggered cell death, apoptosis induced by stress stimuli, and apoptosis resulting from a lack of growth factors, while simultaneously fostering cell growth and proliferation [41,42]. In this regard, Asm inhibitors show potential in providing protection against apoptosis and neurodegeneration, making them promising candidates for the treatment of a range of disorders. These disorders include brain ischemia, stroke, neuronal cell death, Alzheimer’s disease, Parkinson’s disease, Chorea Huntington, spinal cord injuries, seizure disorders, glaucoma, and neurodegeneration in multiple sclerosis [43,44,45,46,47,48]. Hence, the present study tested whether Asm and ceramide signaling contributes to nicotine-induced Nlrp3 inflammasome activation and podocyte injury.

## 2. Materials and Methods

Cell culture: We utilized a mouse podocyte cell line that was conditionally immortalized for our research (kindly provided by Dr. Klotman PE, Division of Nephrology, Mount Sinai School of Medicine, New York, NY, USA) [34,49]. We cultured the cells on collagen I-coated flasks or plates in RPMI 1640 medium, supplemented with recombinant mouse interferon-γ, at a temperature of 33 °C. After a differentiation period at 37 °C for 10–14 days without interferon–γ, the podocytes were utilized for the intended experiments. Podocytes were pretreated with 10 μM amitriptyline (Sigma-Aldrich, St. Louis, MO, USA) for 30 min prior to overnight nicotine treatment (8 μM). Previously, it was shown that nicotine causes podocyte injury between 4 µm to 10 µm, which is equivalent to smoking cigarettes 5–10 times/day [13,50,51]. Hence, the nicotine 8 μM dose was chosen based on our preliminary experiments and previous studies [13,50,51].

### 2.1. Indirect Immuno-Fluorescent Staining and Confocal Microscopy

To investigate the colocalization of inflammasome components in podocytes, cultured cells were fixed with 4% paraformaldehyde (PFA) for 15 min. Following fixation, cells were washed with phosphate-buffered saline (PBS) and incubated overnight at 4 °C with specific primary antibodies targeting key inflammasome and podocyte markers. These included goat anti-NLRP3 (1:100, Abcam, Cambridge, MA, USA) in combination with either rabbit anti-ASC (1:100, Enzo, Plymouth Meeting, PA, USA), rabbit anti-caspase-1 (1:100, Abcam, Cambridge, MA, USA), rabbit anti-podocin (1:200), rabbit anti-desmin (1:100), rabbit anti-ceramide (1:100), or rabbit anti-Asm (1:100) antibodies. Subsequently, the slides were washed, and the primary antibody-labeled cells were then incubated with Alexa-488- or Alexa-555-labeled secondary antibodies for 1 h at room temperature. Following this, the slides were mounted with a DAPI-containing mounting solution and examined using a confocal laser scanning microscope (Leica, Wetzlar, Germany). Images were captured, and the colocalization of NLRP3 with ASC or caspase-1 was analyzed using Image Pro Plus 6.0 software (Media Cybernetics, Bethesda, MD, USA). The data were expressed as the Pearson correlation coefficient (PCC), as previously described [52].

### 2.2. Western Blot Analysis

The protein extracted from cell lysate was separated using SDS-PAGE gel and then transferred onto a polyvinylidene difluoride membrane, which was subsequently blocked. Next, the membrane was incubated with primary antibodies against Podocin, Desmin at a dilution of 1:500 overnight at 4 °C. Afterward, the membrane was incubated with horseradish peroxidase-labeled IgG secondary antibodies. The immunoreactive bands were detected using chemiluminescence methods and visualized on Odyssey chemiluminescence system (Li-Cor Inc., Lincoln, NE, USA). To ensure accurate normalization, the membrane was then re-probed with β-Actin antibody, which served as a loading control. The intensity of the bands was quantified using densitometry with the aid of ImageJ software version 1.44p (NIH, Bethesda, MD, USA).

### 2.3. Caspase-1 Activity and IL-1β Production

Following the treatment with nicotine and the Asm inhibitor, the podocytes were harvested, and their proteins were extracted through homogenization to assess caspase-1 activity using a commercial assay (Biovision, Exton, PA, USA). The obtained data was presented as a fold change relative to the control group, enabling us to assess the impact of the treatments on caspase-1 activity [18]. Additionally, the cell supernatant was collected to measure IL-1β production. For this purpose, a mouse IL-1β ELISA kit from Bender Medsystems, Burlingame, CA, was employed, following the protocol provided by the manufacturer.

### 2.4. Asm Activity Assay

The ASM activity test was performed to assess the enzymatic activity of acid sphingomyelinase (ASM) in the experimental samples. The assay utilized the commercially available Acid Sphingomyelinase assay kit (Abcam, catalog number ab252889) to measure the hydrolysis of sphingomyelin to ceramide and phosphocholine by the ASM enzyme at pH 5.0 [53]. Cell lysates were obtained from cultured podocytes, and the ASM enzyme was extracted. Following the preparation of samples and standards, a reaction mix was added to each well and incubated at 37 °C for 30 min. The absorbance was measured at 570 nm to quantify the product formed during the enzymatic reaction. The ASM activity levels were then determined based on these measurements and expressed as relative enzymatic activity compared to control samples.

### 2.5. Cell Permeability Assay

Podocytes were cultured in the upper chambers of 0.4 μm polycarbonate transwell filters housed in a 12-well filtration microplate (Corning., Glendale, AR, USA). Once the podocytes attained confluence, the existing culture medium was replaced with fresh serum-free RPMI 1640 medium supplemented with 8 μM nicotine with or without the addition of an Asm inhibitor. The following day, the media in the upper chambers were substituted with fresh phenol red-free RPMI 1640, and 70 kDa FITC-dextran (2.5 μmol/L) was added to the top chambers, followed by an incubation period of 2.5 h. After the incubation, the filtration microplate was removed, and the medium from the lower compartment was collected for analysis. The fluorescence intensity was then measured using a spectrofluorimeter with excitation at 494 nm and emissions at 521 nm. The obtained relative permeable fluorescence intensity served as a representation of the cell permeability in response to the different treatments [54].

### 2.6. RNA Interference of Asm

Small interference RNAs (siRNAs) for Asm were commercially obtained from Santacruz Biotechnology, USA. The sequence for Asm siRNA was confirmed to be effective in silencing the Asm gene by the company. siRNA (40 nM) was transfected with the silentfect Lipid Reagent (Bio-Rad, Hercules, CA, USA) as per the manufacturer’s instructions. RNA interference on the expression of the targeted proteins was examined by immunoblotting using anti-Asm antibodies.

### 2.7. Assessment of Superoxide ($O_{2^{.-}}$) Production

Superoxide ($O_{2^{.-}}$) generation was assessed using electron spin resonance (ESR) spectroscopy in accordance with previously validated methodologies [34,54]. In this approach, proteins extracted from podocytes were reconstituted in a modified Kreb’s–Hepes buffer supplemented with 100 mM deferoxamine and 5 mM diethyldithiocarbamate (both from Sigma-Aldrich). To evaluate NADPH-driven $O_{2^{.-}}$ production, 20 µg of protein was combined with 1 mM NADPH and incubated at 37 °C for 15 min, either with or without 200 U/mL superoxide dismutase (SOD). After incubation, the reaction was supplemented with 1 mM of the superoxide ($O_{2^{.-}}$)-specific spin trap, 1-hydroxy-3-methoxycarbonyl-2,2,5,5-tetramethylpyrrolidine (CMH, Noxygen, Elzach, Germany). The sample was promptly transferred into glass capillaries and examined for superoxide ($O_{2^{.-}})$ production kinetics over a 10 min duration using a Miniscope MS5000 ESR spectrometer (Magnettech Ltd., Berlin, Germany). NADPH-driven superoxide ($O_{2^{.-}}$) production was measured and represented as fold changes relative to the control group [54].

### 2.8. Apoptotic Index Quantification Using Flow Cytometry

Murine podocytes were exposed to nicotine with or without amitriptyline, overnight. Following treatment, cells were detached using trypsin, collected, and analyzed for early and late apoptotic populations. Supernatants were also collected along with trypsinized cells to assess necrotic populations. For all treatment groups, cells were resuspended in 1X Annexin V binding buffer (Abcam, Cambridge, CA, USA) and stained with Annexin V-FITC and propidium iodide (Abcam, Cambridge, CA, USA). The stained samples were incubated in the dark at room temperature for 5 min before being transferred into sterile flow tubes equipped with 35 µm strainer caps (Southern Labware, Cumming, GA, USA). Apoptotic and necrotic cell populations were then analyzed using a flow cytometer (Accuri C6 Plus cytometer). The percentage of apoptotic cells (early apoptotic + late apoptotic + necrotic populations) were quantified as fold change relative to the control group.

### 2.9. Statistical Analysis

Data are provided as arithmetic means ± SEM; n represents the number of independent experiments. All data were tested for significance using Student’s unpaired *t*-test or ANOVA followed by a Tukey test as a post hoc test. Only results with *p* < 0.05 were considered statistically significant.

## 3. Results

### 3.1. Inhibition of Asm Attenuated Nicotine-Induced Ceramide Production and Asm Activity

**Figure 1:**
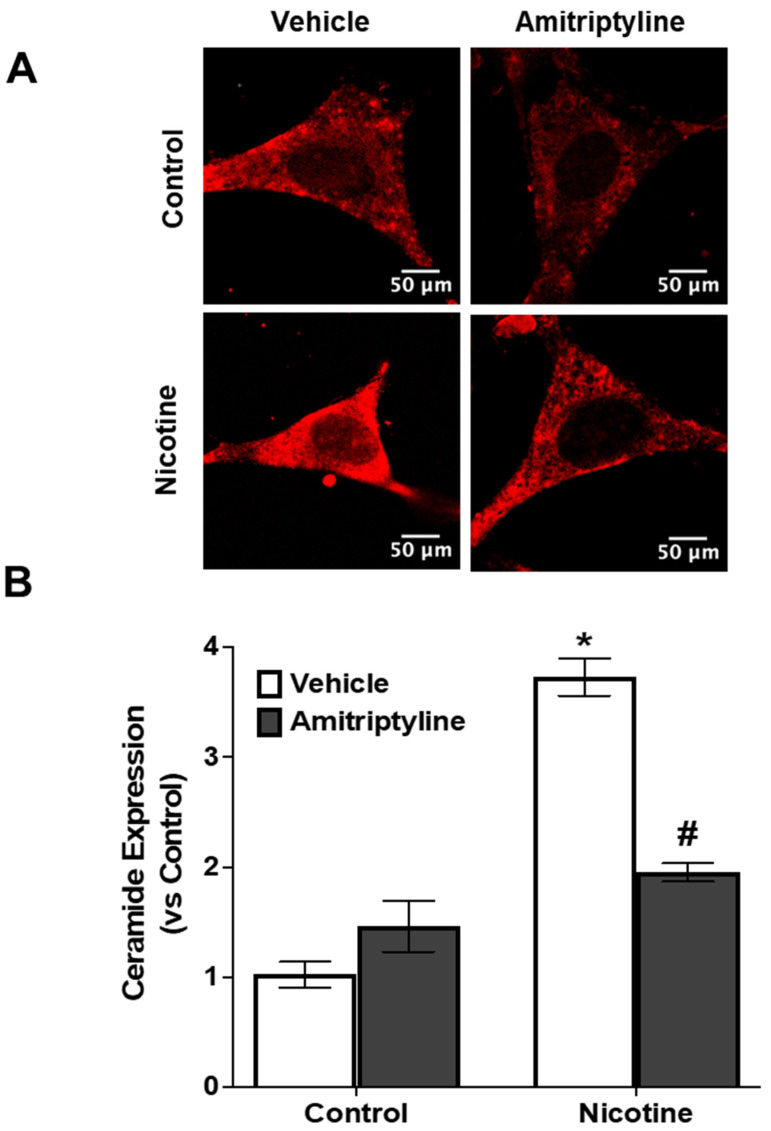
Asm inhibition attenuated nicotine-induced ceramide production. Representative immunofluorescence images (**A**) and quantification analysis (**B**) depicting ceramide expression in podocytes under different treatment conditions, including nicotine stimulation and/or amitriptyline, an Asm inhibitor. Image quantification was performed using ImageJ software. N = 20. * Indicates a significant difference compared to the control group, while # denotes a significant difference from the nicotine-treated group.

**Figure 2:**
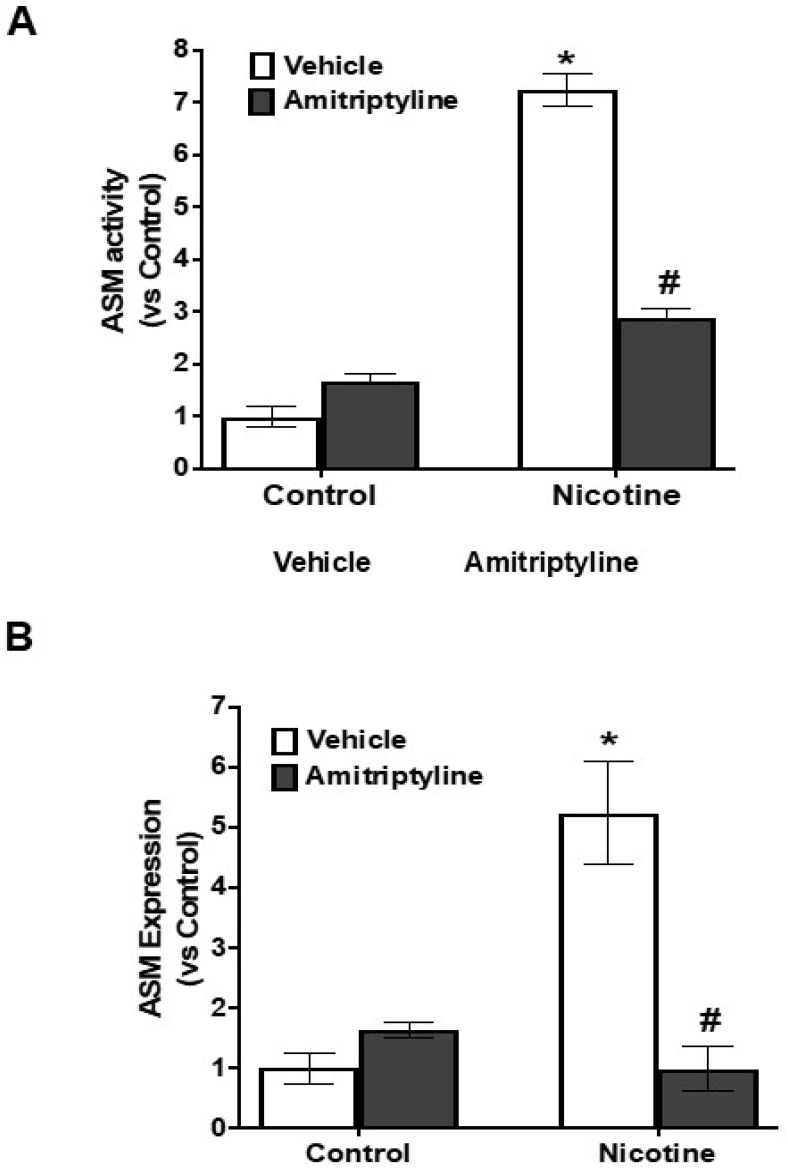
Asm inhibition attenuated nicotine-induced Asm expression and activity. Analysis of acid sphingomyelinase (Asm) activity (**A**) and expression (**B**) in podocytes treated with nicotine and/or amitriptyline, an Asm inhibitor. Immunofluorescence images were quantified using ImageJ software. N = 20 for immunofluorescence analysis. * Represents a significant difference from the control group, while # indicates a significant difference from the nicotine-treated group.

Using confocal microscopy, we observed that nicotine (8 µM, 16 h) treatment increased the ceramide expression (+423%, *p* < 0.001) compared to control cells (Figure 1A,B). However, when podocytes were pretreated with amitriptyline, an Asm inhibitor, the nicotine-induced ceramide production decreased (−253%, *p* < 0.001) (Figure 1A,B). Next, we tested whether nicotine increases the Asm expression. As shown in Figure 2, nicotine treatment increased both Asm activity (+526%, *p* < 0.001) (Figure 2A) and Asm expression (+424%, *p* < 0.001) (Figure 2B) in podocytes compared to control cells. Prior treatment with amitriptyline, an Asm inhibitor, decreased the nicotine-induced Asm activity (−198%, *p* < 0.001) and Asm expression (−428%, *p* < 0.001).

### 3.2. ASM Inhibition Diminished the Nicotine-Induced Formation and Activation of the NLRP3 Inflammasome

**Figure 3:**
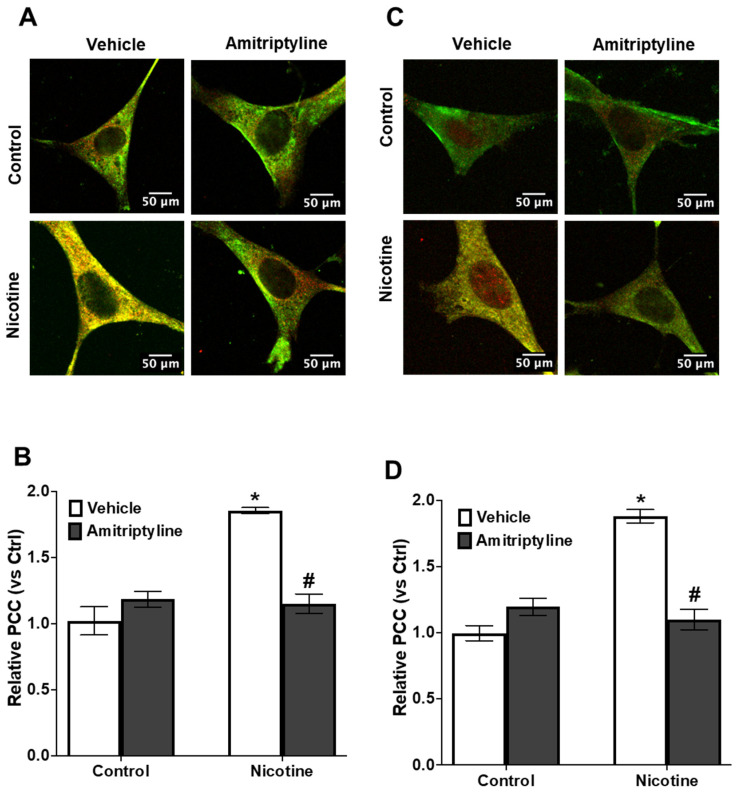
Inhibition of Asm attenuated nicotine-induced inflammasomes formation in podocytes. Confocal images representing the colocalization of Nlrp3 (green) with Caspase-1 (red) (**A**) and Nlrp3 (green) with Asc (red) (**C**) in podocytes (original magnification ×100). Summarized data showing the fold change of the Pearson coefficient correlation (PCC) for the colocalization of Nlrp3 with caspase-1 (**B**) and Nlrp3 with Asc (**D**). Ctrl: control, Veh: vehicle, Ami: amitriptyline. Images were quantified using Image Pro Plus software. N = 18–20. * Significant difference from the control, # Significant difference from the nicotine-treated group.

**Figure 4:**
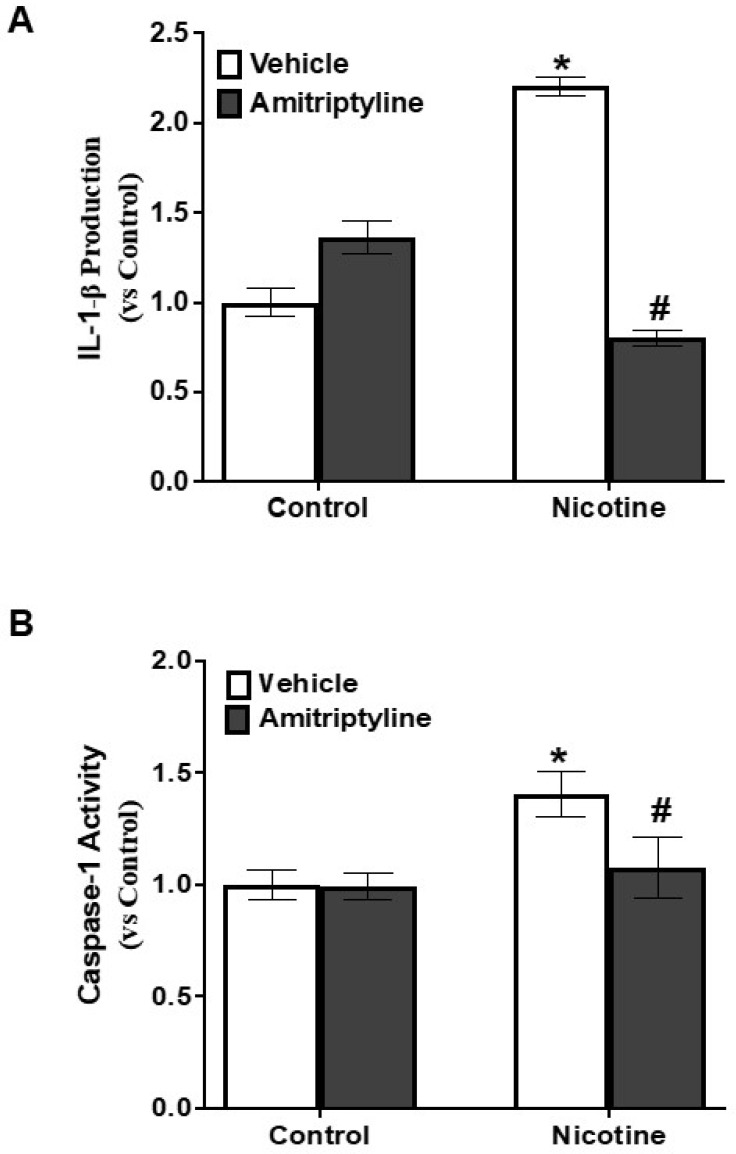
Inhibition of Asm attenuated nicotine-induced inflammasomes activation in podocytes. Data are presented as arithmetic means ± SEM (n = 6 per group) for IL-1β production (**A**) and caspase-1 activity (**B**) in podocytes exposed to nicotine, with or without amitriptyline, an Asm inhibitor. * Significant difference from the control, # Significant difference from the nicotine-treated group.

As illustrated in Figure 3, nicotine treatment enhanced the co-localization of NLRP3 with Caspase-1 (+37%, *p* < 0.05) (Figure 3A,B) and Nlrp3 with ASC (+78%, *p* < 0.001) (Figure 3C,D), as evidenced by an increase in yellow fluorescence staining in podocytes. However, pretreatment with Asm inhibitor amitriptyline decreased the nicotine-induced co-localization of Nlrp3 with Asc (−45%, *p* < 0.05) and Nlrp3 with caspase-1 (−17%, *p* < 0.05). Further, we also observed that nicotine treatment increases the IL-1β production (+84%, *p* < 0.001) (Figure 4A) and caspase-1 activity (+36%, *p* < 0.001) (Figure 4B) when compared to the control cells. However, when podocytes were pretreated with an Asm inhibitor, amitriptyline, we found a reduction in nicotine-induced IL-1β production (−93%, *p* < 0.001) and caspase-1 activity (−32%, *p* < 0.05). In addition, we also found that Asm siRNA transfection attenuates the nicotine-induced IL-1 β production (−83%, *p* < 0.05) (Supplemental Figure S1).

### 3.3. Inhibiting ASM Safeguards Podocytes Against Damage Caused by Nicotine

**Figure 5:**
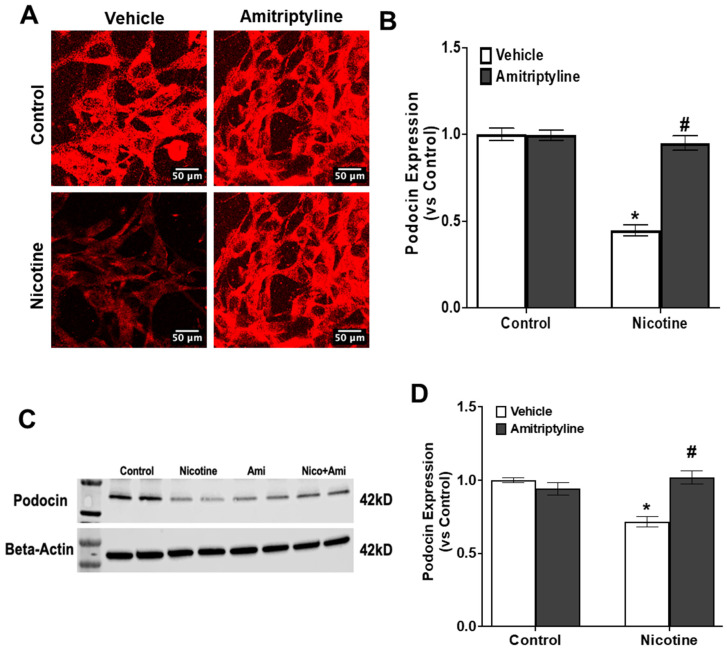
Inhibition of Asm attenuated nicotine-induced podocytes damage. Confocal images represent the expressions of Podocin (**A**) and summarized quantification of Podocin (**B**). Western Blot data show the expression of Podocin (**C**) and summarized quantification of Podocin (**D**). N = 15–20 each group for immunofluorescence expression. Ctrl: control, Veh: vehicle, Ami: amitriptyline. Image analysis was performed using ImageJ software. * Indicates a significant difference from the control group, while # denotes a significant difference from the nicotine-treated group.

**Figure 6:**
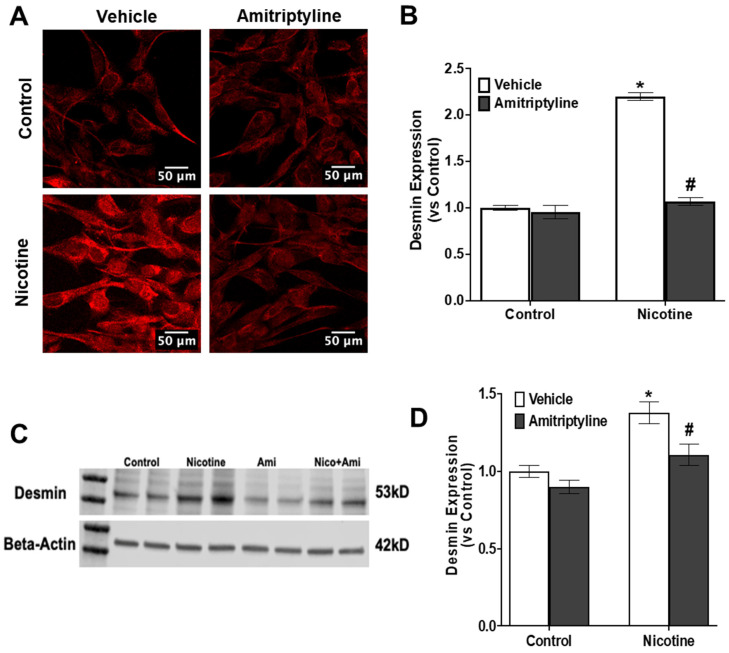
Inhibition of Asm attenuated nicotine-induced podocytes damage. Confocal images represent the expressions of desmin (**A**) and summarized quantification of desmin (**B**). Western blot data show the expression of desmin (**C**) and summarized quantification of desmin (**D**). N = 15–20 each group for immunofluorescence expression. Ctrl: control, Veh: vehicle, Ami: amitriptyline. Images were quantified using Image J software. * Significant difference from the control; # significant difference from the nicotine-treated group.

To evaluate the degree of damage to podocytes, we monitored the protein expression of crucial slit diaphragm molecules, specifically podocin (Figure 5A–D) and desmin (Figure 6A–D). Podocin, being a podocyte-specific marker, tends to decrease in expression during injury, whereas desmin, a marker of podocyte damage, increases under such conditions. Utilizing techniques such as immunofluorescence analysis and Western blot analysis, we investigated the effects of nicotine treatment and Asm inhibitor on podocytes. Our findings revealed that nicotine-treated podocytes exhibited a significant reduction in podocin staining (−55%, *p* < 0.05), indicating podocyte damage, along with an increase in desmin staining (+120%, *p* < 0.001), further confirming the occurrence of podocyte injury. However, prior treatment with an Asm inhibitor showed promising results in protecting podocytes from such damage. The Asm inhibitor pretreatment led to a preservation of podocin (+123%, *p* < 0.001) and desmin protein expression (−105%, *p* < 0.001), which returned to levels similar to those observed in the control group. These results suggest that the Asm inhibitor may offer protection against podocyte damage induced by nicotine, highlighting its potential as a therapeutic agent in preserving podocyte function.

### 3.4. ASM Inhibitor Prevented the Increased Cell Permeability in Podocytes Induced by Nicotine

**Figure 7:**
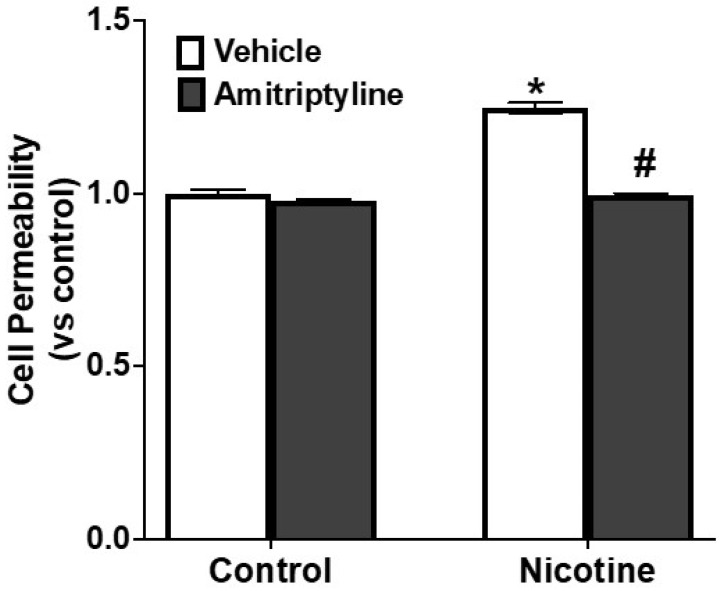
Suppression of Asm effectively reduced nicotine-induced increases in podocyte permeability. Data are presented as arithmetic means ± SEM (n = 9 per group) for podocyte permeability in podocytes with or without nicotine stimulation and/or amitriptyline, an Asm inhibitor. * Indicates a significant difference from the control group, while # denotes a significant difference from the nicotine-treated group.

Additionally, we investigated the impact of NLRP3 inflammasome activation on podocyte monolayer integrity and its role in mediating nicotine-induced alterations in barrier function. Nicotine treatment resulted in an increase in podocyte monolayer permeability (+36%, *p* < 0.05) when compared to control cells (Figure 7). On the other hand, blocking Asm prior to nicotine exposure effectively preserved podocyte monolayer integrity and prevented permeability (−36%, *p* < 0.05). These findings suggest that the amitriptyline blocks the disruption of podocyte monolayers associated with nicotine through its influence on Nlrp3 inflammasomes.

### 3.5. Nicotine-Induced Podocyte Damage Requires ROS

**Figure 8:**
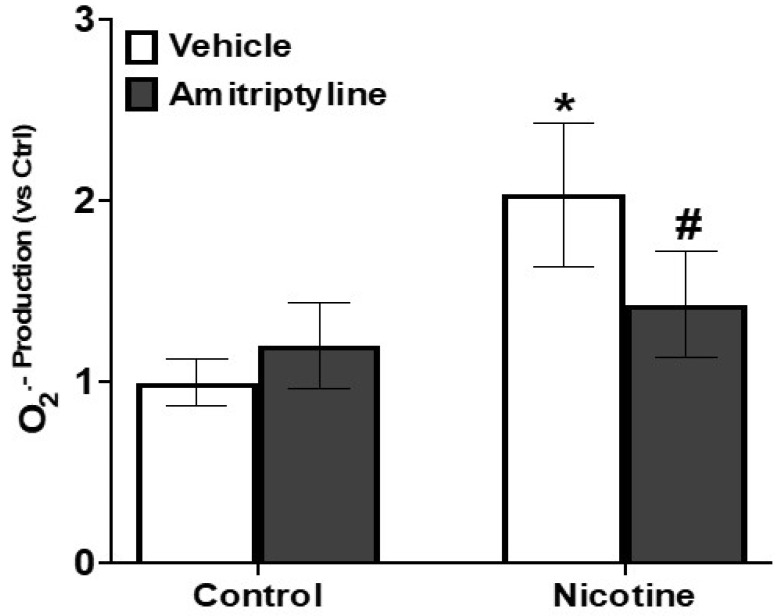
O_2_.^−^ Production in podocytes with or without nicotine and/or amitriptyline treatment. Values are arithmetic means ± SE (n = 6 each group) of O_2_.^−^ production in podocytes with or without nicotine and/or amitriptyline treatment. Ctrl: control, * significant difference (*p* < 0.05) compared to the control group; # significant difference from the nicotine-treated group.

The involvement of reactive oxygen species (ROS) in NLRP3 inflammasome activation has been extensively documented across various cell types [14,17]. Therefore, we examined whether nicotine stimulates $O_{2^{.-}}$ production in podocytes compared to control cells. Our findings revealed that nicotine treatment markedly increased $O_{2^{.-}}$ production (+103%, *p* < 0.05) compared to control cells. In contrast, pretreatment with amitriptyline decreased the nicotine-induced superoxide (O_2_.^−^) production (−42%, *p* < 0.05) (Figure 8).

### 3.6. Inhibiton of Asm Recovers Nicotine-Induced Podocyte Apoptosis

**Figure 9:**
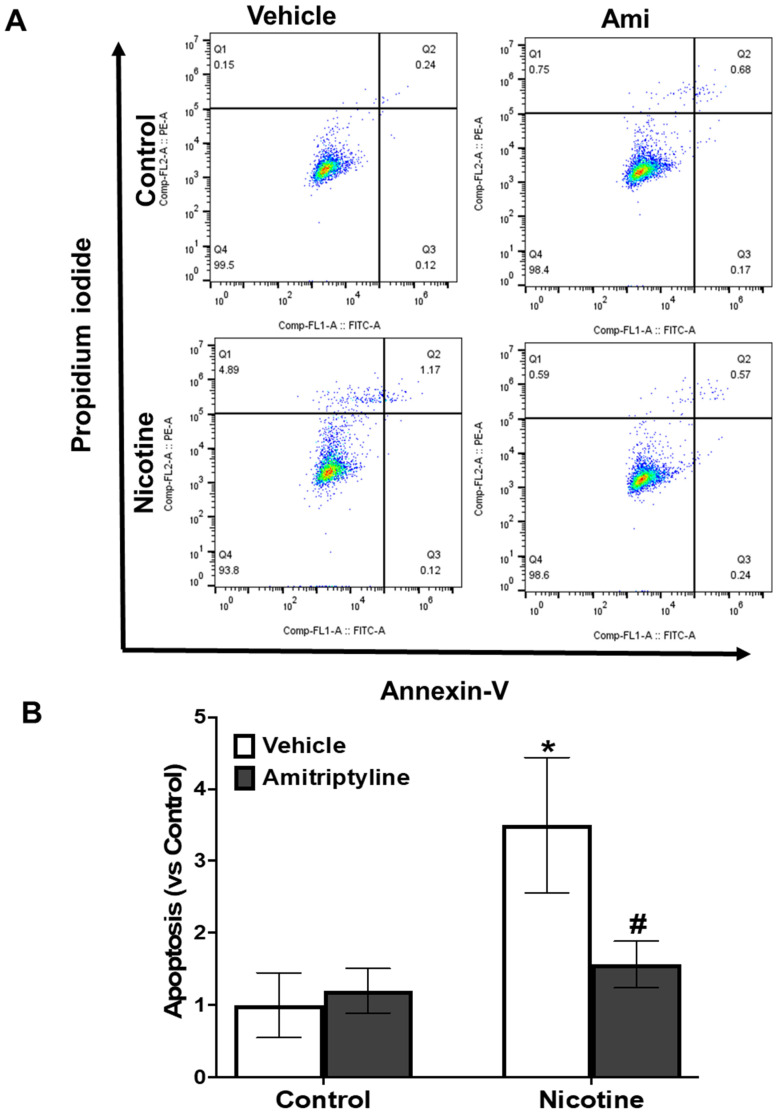
Inhibition of Asm protects against nicotine-induced apoptosis in podocytes. Flow cytometry analysis (**A**) and corresponding quantification (**B**) were performed to investigate the role of Asm in nicotine-mediated podocyte apoptosis. Data were analyzed using FlowJo v10.10.0 software, with apoptotic cells (%) calculated as the sum of early apoptotic, late apoptotic, and necrotic populations. Results are presented as fold change relative to the control group. * *p* < 0.05 vs. control group, *p* < 0.05 vs. nicotine-treated group; ^#^ significant difference from the nicotine-treated group. Q1: necrotic cells, Q2: late apoptotic cells, Q3: early apoptotic cells, Q4: live cells, Ctrl: control podocytes, Nico: nicotine (8 µM)-treated podocytes.

We examined the effect of nicotine on apoptosis in murine podocytes, both in the presence and absence of an Asm inhibitor, amitriptyline. Flow cytometry analyses revealed that nicotine exposure led to significant increases in necrotic (Q1) and late apoptotic (Q2) cell populations when compared to vehicle-treated podocytes (Figure 9A,B). Conversely, pretreatment with amitriptyline effectively mitigated the nicotine-induced elevation in apoptotic cell populations (Figure 9A,B). These findings collectively indicate that nicotine-driven apoptosis in podocytes is Asm-dependent, and pharmacological inhibition of Asm with amitriptyline provides protective effects against nicotine-induced podocyte apoptosis.

## 4. Discussion

The present study was designed to explore the role of acid sphingomyelinase (Asm), a ceramide-producing enzyme, in nicotine-induced NLRP3 inflammasome activation and podocyte injury. We found that nicotine treatment enhanced the Asm activity and ceramide production, which was attributed to NLRP3 inflammasome activation in podocytes and ultimately led to podocyte injury. Inhibition of Asm prevented nicotine-induced Nlrp3 inflammasome activation and subsequent podocyte injury. The findings for the first time demonstrate the critical role of Asm in the activation of Nlrp3 inflammasomes and subsequent podocyte injury associated with smoking.

Inflammasomes are crucial components of the innate immune system that respond to pathogenic or danger signals by initiating the activation of inflammatory processes [55]. Inflammasomes are multiprotein complexes, composed of different sensor proteins like AIM2, Pyrin, and Nlrp3. They have the capacity to detect intracellular disruptions that initiate the cleavage of proinflammatory cytokines such as interleukin-1β (IL-1β), HMGB1 [56], and interleukin-18 (IL-18) [57]. Inflammasomes activation is strongly regulated and plays a fundamental role in maintaining immune homeostasis [58]. The dysregulation of inflammasome activation has been associated with the development of several inflammatory and autoimmune diseases [59]. Nlrp3 inflammasomes are the most studied and important in the broad range of mammalian cells among the several types of inflammasomes. Notably, it is particularly abundant in podocytes located within the glomeruli of the kidney [34]. The development of diseases linked to Nlrp3 inflammasome activation underscores the vital role played by this multi-protein complex in instigating inflammatory responses [60]. It is composed of several proteins, including the Nlrp3 sensor protein, the adaptor protein ASC (apoptosis-associated speck-like protein containing a CARD), and the effector protein caspase-1 [26]. The dysregulated activation of Nlrp3 has been implicated in a range of conditions, including autoinflammatory syndromes, metabolic disorders, and neurodegenerative diseases [61]. The sustained activation of Nlrp3 inflammasomes leads to excessive production of pro-inflammatory cytokines like interleukin-1β (IL-1β) and interleukin-18 (IL-18), contributing to tissue damage and chronic inflammation [62]. Inflammatory conditions such as gout, type 2 diabetes, obesity, atherosclerosis, and Alzheimer’s disease exhibit aberrant Nlrp3 activation as a key contributor to disease pathogenesis [19,23,25,61]. Recent studies have shown that nicotine contributes to the formation and activation of the Nlrp3 inflammasome in podocytes [13]. However, it remains unknown how nicotine activates the Nlrp3 inflammasomes and contributes to podocyte injury or dysfunction. A comprehensive understanding of the underlying mechanisms linking Nlrp3 inflammasome activation to disease progression is essential for the development of targeted therapeutic interventions. In this regard, Ceramide accumulation can trigger pro-inflammatory responses, oxidative stress, and apoptotic pathways, collectively contributing to renal cell damage and dysfunction [63]. Ceramide production primarily occurs through two main pathways. One pathway involves the breakdown of membrane sphingomyelin through the actions of different sphingomyelinases, including acid sphingomyelinase (Asm) and neutral sphingomyelinase (NSM). These enzymes hydrolyze sphingomyelin to yield ceramide. The other pathway involves de novo synthesis, initiated by serine palmitoyl transferase (SPT), which catalyzes the condensation of serine and palmitoyl-CoA to form 3-ketosphinganine. Subsequent steps involving ceramide synthase lead to the production of ceramide [64]. These pathways collectively regulate ceramide levels, influencing a diverse array of cellular processes and functions. Elevated ceramide levels have been associated with numerous disease pathways, contributing to the progression of various disorders [65,66]. Ceramide’s role extends to cardiovascular health, where it promotes inflammation, endothelial dysfunction, and atherosclerosis, contributing to the pathogenesis of cardiovascular diseases [66,67,68]. Moreover, ceramide-mediated disruptions in mitochondrial function and apoptotic pathways have implications for neurodegenerative disorders like Alzheimer’s disease. Ceramide has significant effects on many diseases, highlighting its important role in impacting various disease processes. In this context, our current study tested whether the Asm mediates nicotine-induced Nlrp3 inflammasomes activation and contributes to podocyte injury. We found that nicotine induced ceramide production and Asm expression in podocytes. However, prior treatment with amitriptyline, an Asm inhibitor, blocked nicotine-induced ceramide production and Asm expression. Collectively, these findings indicate that the increase in ceramide levels induced by nicotine is primarily driven by Asm.

Emerging evidence suggests that ceramide activation plays a crucial role in triggering caspase-1 activation and IL-1β production via the NLRP3 inflammasome pathway, contributing to pathological conditions like obesity, podocyte damage, and acute lung injury [14,25,69,70]. Indeed, our findings reveal that nicotine-driven ceramide production plays a key role in promoting NLRP3 inflammasome assembly in podocytes, as reflected by the colocalization of Nlrp3 with ASC or Nlrp3 with caspase-1 (Figure 3). These colocalizations were substantially blocked by amitriptyline treatment. Biochemical assessments demonstrated that nicotine exposure led to a significant rise in caspase-1 activity and IL-1β production in podocytes compared to untreated controls. Notably, pretreatment with amitriptyline effectively counteracted these effects, preventing nicotine-induced increase in inflammasome activation (Figure 4), suggesting the pivotal role of acid sphingomyelinase in mediating the nicotine-induced inflammasome activation in podocytes.

Next, we tested the activation of Nlrp3 inflammasomes induced by nicotine, leading to podocyte injury or dysfunction, which could be mitigated by acid sphingomyelinase. Podocytes, which are specialized epithelial cells lining the outermost layer of glomeruli, have a pivotal role in ensuring the appropriate filtration of substances in the kidney. Injury to these podocytes signifies compromises glomerular filtration, which can ultimately progress to glomerular sclerosis [71]. Importantly, a clear indication of podocyte damage is the extension of the foot-processing, a phenomenon often accompanied by disruptions in the actin cytoskeleton; elevated expression of desmin, an indicator of podocyte injury; and diminished levels of podocin, a critical molecule for cell polarity and survival. Our study revealed that nicotine stimulation led to reduced podocin expression and increased desmin expression in podocytes in both immunofluorescence and Western blot analyses. However, intriguingly, the inhibition of inflammasome activation using an Asm inhibitor was associated with the preservation of podocyte morphology. This preservation was demonstrated by the maintenance of well-defined F-actin fiber arrangements, a functional consequence highlighted by the sustained expression of podocin and the prevention of increased desmin expression. This suggests that inhibiting inflammasome activation using an amitriptyline holds promise not only in preserving the structural integrity of podocytes but also in maintaining their functional attributes, potentially serving as a therapeutic avenue to counteract nicotine-induced podocyte damage.

Further, we explored how an Asm inhibitor could counteract the heightened permeability of podocyte layers caused by nicotine. It is worth noting that our results showed that podocytes exposed to nicotine experienced a noticeable increase in the permeability of these layers. This finding is particularly significant because there is substantial evidence indicating that oxidative stress in the glomeruli contributes to increased permeability of these epithelial layers, which is often observed in various health issues like diabetes, nephritis, hypertension, and hyperhomocysteinemia (hHcys) [72,73]. This observed increase in cellular permeability in the nicotine groups is crucial in the context of glomerular health [69], as it constitutes a key contributor to the progression of glomerular injury and eventual sclerosis [54]. The intricate connection between oxidative stress, compromised cellular barriers, and the development of renal damage underscores the significance of our investigation in evaluating the potential of Asm to mitigate the nicotine-induced increase in the monolayer permeability of podocytes. By focusing on this critical aspect of podocyte function, our study offers insights into the mechanisms underpinning glomerular dysfunction and offers a potential avenue for therapeutic interventions aimed at preserving podocyte integrity.

To further explore the mechanism of nicotine-induced Nlrp3 inflammasome activation in podocytes, we determined the NADPH oxidase-derived O_2_·^−^ production in podocytes with or without stimulation of nicotine and/or an Asm inhibitor, amitriptyline. It is well documented that ASM- and ceramide-associated MR clustering and related products such as O_2_^•−^ may act as redox-signaling messengers to activate the Nlrp3 inflammasome, which serves as the bridging and amplifying mechanism leading to a robust inflammatory response that eventually progresses to podocyte injury [74]. Indeed, the present study showed that nicotine significantly increased the NADPH oxidase-dependent O_2_·^−^ production in podocytes compared to control cells. However, prior amitriptyline treatment attenuated the nicotine-induced increase in superoxide production. These results suggest that NADPH oxidase activation by nicotine and subsequent production of superoxide are upstream of Nlrp3 inflammasome activation, given that inhibition of Asm significantly decreased the levels of NADPH oxidase-derived superoxide and inflammasome activation.

It has been reported that depletion of podocytes in the glomeruli is the strongest predictor for the progression of glomerulosclerosis, where fewer cells predict more rapid progression [75]. Recently, it has been demonstrated that podocyte apoptosis is a key mechanism leading to podocyte loss in diabetic nephropathy [76], PAN-induced nephrosis [77], and transgenic mice expressing transforming growth factor-β1 (TGF-β1) [78]. Our flow cytometric analysis showed that nicotine treatment increased the rate of podocyte apoptosis compared to vehicle-treated cells, which were blocked or attenuated by the inhibition of Asm. Since podocytes are terminally differentiated cells in vivo with a limited capacity to proliferate, it seems that Asm and the consequent activation of NADPH oxidase importantly contribute to podocyte loss and subsequent glomerulosclerosis associated with smoking through its action to induce apoptosis. Despite these encouraging results, the current study suffers from some limitations: 1) We applied in vitro podocyte cell lines to test our hypothesis; therefore, our conclusions and interpretations are valid only for in vitro effects, whereas additional studies are required in addition to explore whether a similar behavior is present in in vivo studies. 2) We utilized only one cell line of glomerular cells, namely podocytes; therefore, the application of additional glomerular cell lines is appealing.

In conclusion, our study demonstrated that the Asm inhibitor amitriptyline attenuated nicotine-triggered NLRP3 inflammasome activation and its resulting podocyte damage. Targeting acid sphingomyelinase (Asm) by inhibiting its activity may be a novel therapeutic strategy for the treatment and prevention of smoking-induced kidney diseases.

## Data Availability

The data presented in this study are available from the corresponding author upon request.

## Supplementary Materials

The following supporting information can be downloaded at https://www.mdpi.com/article/10.3390/biomedicines13020416/s1, Figure S1. Asm siRNA transfection attenuated nicotine induced inflammasomes activation in podocytes. Values are arithmetic means ± SEM (n = 4–7 each group) of IL-1β production in podocytes with or without stimulation of nicotine and/or an Asm siRNA transfection.
