# Supplementary material for: Acid Sphingomyelinase and Ceramide Signaling Pathway Mediates Nicotine-Induced NLRP3 Inflammasome Activation and Podocyte Injury
Source: Biomedicines. 2025 Feb 9;13(2):416. doi: 10.3390/biomedicines13020416 (PMC11852453; doi:10.3390/biomedicines13020416)
Supplement: Supplementary file 1 [file biomedicines-13-00416-s001.zip › biomedicines-3421036-supplementary.pdf]

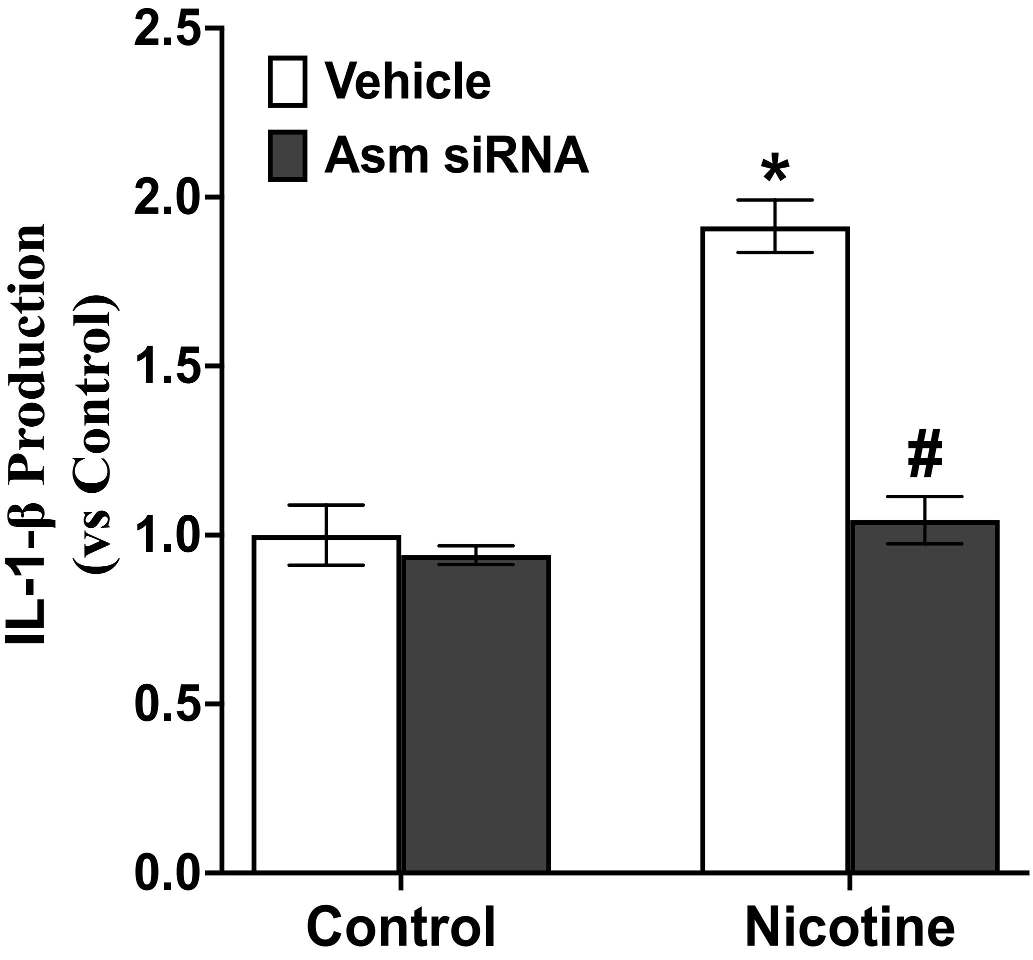

Supplemental Figure 1. Asm siRNA transfection attenuated nicotine induced inflammasomes activation in podocytes. Values are arithmetic means ± SEM (n=4-7 each group) of IL-1β production in podocytes with or without stimulation of nicotine and/or an Asm siRNA transfection.
